# Nocturnal Hypoxia Improves Glucose Disposal, Decreases Mitochondrial Efficiency, and Increases Reactive Oxygen Species in the Muscle and Liver of C57BL/6J Mice Independent of Weight Change

**DOI:** 10.1155/2018/9649608

**Published:** 2018-02-04

**Authors:** Simona Ioja, Srikanth Singamsetty, Catherine Corey, Lanping Guo, Faraaz Shah, Michael J. Jurczak, Bryan J. McVerry, Sruti Shiva, Christopher P. O'Donnell

**Affiliations:** ^1^Division of Endocrinology and Metabolism, University of Pittsburgh Medical Center, University of Pittsburgh, 3459 Fifth Avenue, 628 NW, Pittsburgh, PA 15213, USA; ^2^Division of Pulmonary, Allergy and Critical Care Medicine, Department of Medicine, University of Pittsburgh Medical Center, University of Pittsburgh, 3459 Fifth Avenue, 628 NW Pittsburgh, PA 15213, USA; ^3^Department of Pharmacology and Chemical Biology, University of Pittsburgh Medical Center, University of Pittsburgh, 3459 Fifth Avenue, 628 NW Pittsburgh, PA 15213, USA

## Abstract

Although acute exposure to hypoxia can disrupt metabolism, longer-term exposure may normalize glucose homeostasis or even improve glucose disposal in the presence of obesity. We examined the effects of two-week exposure to room air (Air), continuous 10% oxygen (C10%), and 12 hr nocturnal periods of 10% oxygen (N10%) on glucose disposal, insulin responsiveness, and mitochondrial function in lean and obese C57BL/6J mice. Both C10% and N10% improved glucose disposal relative to Air in lean and obese mice without evidence of an increase in insulin responsiveness; however, only the metabolic improvements with N10% exposure occurred in the absence of confounding effects of weight loss. In lean mice, N10% exposure caused a decreased respiratory control ratio (RCR) and increased reactive oxygen species (ROS) production in the mitochondria of the muscle and liver compared to Air-exposed mice. In the absence of hypoxia, obese mice exhibited a decreased RCR in the muscle and increased ROS production in the liver compared to lean mice; however, any additional effects of hypoxia in the presence of obesity were minimal. Our data suggest that the development of mitochondrial inefficiency may contribute to metabolic adaptions to hypoxia, independent of weight, and metabolic adaptations to adiposity, independent of hypoxia.

## 1. Introduction

Hypoxia is a common consequence of pathology or disease, but also induces adaptive physiologic responses with altitude exposure [1, 2]. Hypoxia can occur in cells, tissues, and organs and manifest as intermittent or sustained in nature [3]. Consequently, the pattern and duration of hypoxia can impact responses. For example, just a few brief periods of hypoxia and reoxygenation in the myocardium can protect the heart from subsequent infarction [4, 5], whereas brief nocturnal periods of hypoxia and reoxygenation, occurring over years to decades in patients with sleep apnea, can cause significant pathology [6–10]. Alternatively, short-term exposure to altitude hypoxia can induce acute mountain sickness [11], whereas adaptation occurs over days to weeks at altitude [12, 13]. Recently, we have shown that four weeks of sustained hypoxia in lean mice, simulating altitude exposure, improves glucose disposal relative to four weeks of intermittent hypoxia, simulating obstructive sleep apnea [14]. Studies of high-altitude natives provide biologic plausibility for chronic altitude exposure to improve glucose metabolism [15, 16], and, experimentally, ten nights of moderate hypoxic exposure in obese humans improved glucose disposal [17]. It is likely that sustained hypoxic exposure can impact several pathways to increase glucose uptake. Specifically, hypoxia could affect the efficiency of mitochondrial oxidative metabolism; a decrease in the respiratory control ratio (RCR), a measure of the efficiency of the mitochondria to couple oxygen consumption to ATP production, could improve glucose disposal but at the potential cost of increasing reactive oxygen species [17, 18]. To date, studies of chronic hypoxic-induced changes in RCR have focused on skeletal muscle function, rather than metabolism, and report either a decrease [19, 20] or no change in RCR [21].

Although data suggest that acute exposure to hypoxia can induce insulin resistance [22], longer-term exposure to hypoxia may restore [13] or even improve insulin sensitivity [17, 23]. In addition, the mechanisms of metabolic adaptation to hypoxia are potentially impacted by obesity and the presence of a basal state of insulin resistance. Given that mitochondria are impacted by both obesity [24] and hypoxia [25, 26], we examined the metabolic effects of diet-induced obesity and sustained hypoxic exposure on whole body glucose disposal and insulin responsiveness, and mitochondrial function of the muscle and liver in C57BL/6J mice fed either a regular chow or high-fat diet and exposed to continuous or nocturnal hypoxia for two weeks.

## 2. Methods

### 2.1. Ethical Approval and Surgical Procedures

This study was carried out in strict accordance with the recommendations in the *Guide for the Care and Use of Laboratory Animals* of the National Institutes of Health. Animal protocols were approved by the Institutional Animal Care and Use Committee at the University of Pittsburgh (protocol number 12050422).

### 2.2. Protocol

Experiments were conducted in lean 10-week-old male C57BL/6J mice on a regular chow diet (14% fat) and 22-week-old obese C57BL/6J mice fed a high-fat diet for 16 weeks (D12492: 60% kcal% fat; Research Diets, New Brunswick, NJ). Groups of lean and obese mice were exposed to room air (control), nocturnal 10% inspired oxygen for 12 hrs/day during the light cycle (N10%), or continuous 10% inspired oxygen for 24 hrs/day. Animals were acclimated to the experimental cages over a one-week period prior to beginning the hypoxic interventions and handled daily in preparation for conducting the glucose tolerance test (GTT) and insulin tolerance test (ITT). On day 0, prior to hypoxic intervention and experimental group assignment, a GTT was performed after an overnight fast. After collecting a basal plasma sample (*t* = 0), mice were injected intraperitoneally with a 2 g/kg glucose bolus and blood glucose was measured (Prodigy AutoCode glucometer: ∼1 *μ*l whole blood; Diagnostic Devices, Charlotte, NC) by tail bleed at set time points (*t* = 15, 30, 45, 60, 90, and 120 min). On day 1, an ITT was performed after a two-hour fast by administering an intraperitoneal 0.75 U/kg bolus of insulin and measuring blood glucose by tail bleed at *t* = 15, 30, 45, 60, 90, and 120 min. After completion of the ITT, a random block design was used to determine if mice were exposed to room air, N10%, or C10%. On day 14 of exposure, the GTT was repeated and on day 15 the ITT was repeated. Animals were maintained under exposure conditions until they were killed and tissue was harvested on day 16.

### 2.3. Hypoxic Exposure

Mice were housed in regular cages that were customized to deliver hypoxic gas or room air as control [27]. Gas (~3 l·min^−1^) entered the cages from ports evenly spaced near the bottom on all four sides at the level of the bedding material. The cage lid was filled with foam and sealed at the edges, and gas entering through the input ports at the bottom of the cage was exhausted through a vacuum connection in the cage lid. The standard metal grill inside the cage was used to hold food and water supplies.

A gas control delivery system regulated the flow of room air and N_2_ into the customized cages housing the mice. A series of programmable solenoids and flow regulators enabled the stable delivery of 10% inspired oxygen or room air. The use of multiple inputs into the cage produced a uniform inspired oxygen level throughout the cage, which was continuously monitored with an O_2_ analyzer (model OM11, SensorMedics, Yorba Linda, CA).

### 2.4. Mitochondrial Respiration (State 3, State 4, and Respiratory Control Ratio)

Mitochondrial oxygen consumption was measured in fresh homogenates of the gastrocnemius muscle and liver tissue homogenate using a Clark-type oxygen electrode as previously described [28, 29]. Briefly, each tissue (2 mg/ml) was suspended in respiratory buffer (120 mM KCl, 3 mM HEPES, 1 mM EGTA, 25 mM sucrose, 5 mM MgCl_2_, and 5 mM KH_2_PO_4_, pH 7.4); in the sealed respirometer, state 4 respiration was measured after the addition of succinate (5 mm). State 3 respiration rate was measured after the addition of ADP (30 nM). Both state 3 and state 4 respiratory rates were completely inhibited in the presence of rotenone (20 *μ*M). Data were normalized to total tissue protein content, and the respiratory control ratio (RCR) was calculated as the product of state 3/state 4.

### 2.5. Mitochondrial ROS Production

Mitochondrial hydrogen peroxide production was measured by spectrophotometrically monitoring the rotenone-sensitive oxidation of Amplex Red (at 585 nm) by tissue homogenates in the presence of succinate and ADP.

### 2.6. GLUT Expression

GLUT expression in tissue (lysed and sonicated) was measured by Western blot. Antibodies used included GLUT-1 (ab115730), GLUT-2 (ab104622), GLUT-3 (ab41525), and GLUT-4 (ab654) from Abcam (Cambridge, MA). Western blots quantified as relative intensity ratio of GLUT1–4 to *β*-actin housekeeping protein.

### 2.7. Phosphofructokinase Activity

PFK activity was measured in permeabilized tissue using a colorimetric assay kit (Sigma-Aldrich; St. Louis, MO). Briefly, PFK activity was coupled to the conversion of ADP to AMP and NADH, which reduced a colorless probe to a colored product that was detected at 450 nm.

### 2.8. Plasma Free Fatty Acids and Lactate

Plasma fatty acid levels were measured using an *in vitro* enzymatic colorimetric method according to the manufacturer's instructions (Wako Diagnostics, NEFA kit). Plasma lactate levels were measured using an oxygen electrode system (Analox GL5) and reaction with lactate oxidase, where the rate of oxygen consumption is directly proportional to lactate concentration in the sample.

### 2.9. Statistical Analyses

Differences between means in lean and obese mice in each of the three experimental groups (room air, N10%, and C10%) were determined by either one-way or two-way ANOVA. Where the ANOVA indicated statistical significance (*p* < 0.05), differences between the means were determined by a post hoc Tukey test. The use of paired or unpaired *t*-test is reported in figure or table legends where appropriate. Data are reported as mean ± s.e.m.

## 3. Results

### 3.1. Hypoxia Improves Glucose Disposal in Lean and Obese Mice

Under control room air conditions, lean mice on the regular chow diet remained weight stable and obese mice on the high-fat diet increased body weight (Table 1). After exposure to N10%, there was a small increase in body weight in lean mice, whereas the obese mice were weight stable. In contrast, exposure to C10% caused body weight decreases in both lean and obese mice, with greater weight loss occurring in the obese mice. There were no significant differences in feeding between C10% groups on either diet (Table 1).

**Figure 1:**
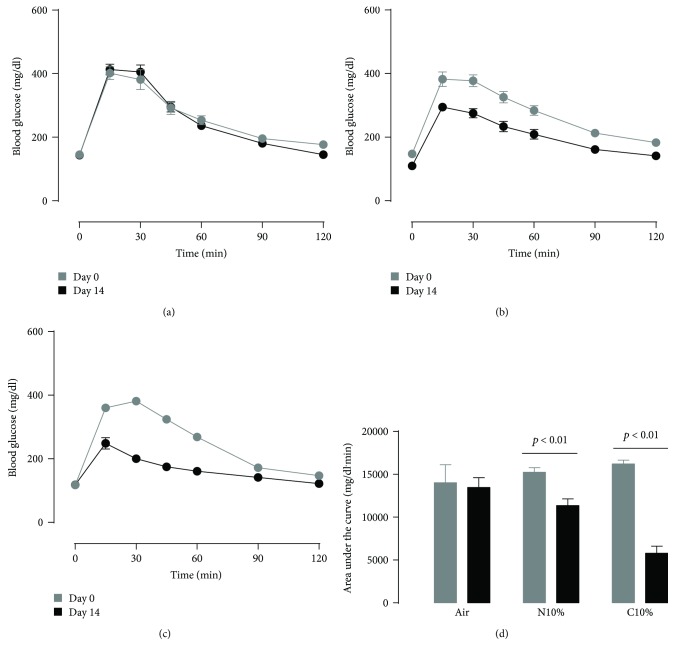
Lean mice: intraperitoneal glucose tolerance test on day 0 (before) and day 14 (after) exposure to two weeks of (a) room air (Air; *n* = 10), (b) nocturnal 10% hypoxia (N10%; *n* = 7), and (c) continuous 10% hypoxia (C10%; *n* = 8) and (d) corresponding mean ± s.e.m. area under the glucose curve. Statistical differences marked by horizontal lines determined by two-tailed paired *t*-test.

**Figure 2:**
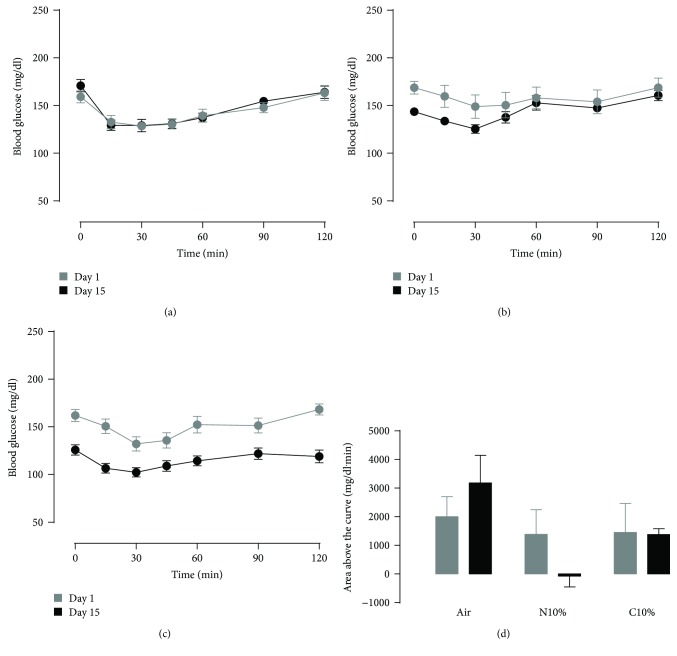
Lean mice: intraperitoneal insulin tolerance test on day 0 (before) and day 14 (after) exposure to two weeks of (a) room air (Air; *n* = 10), (b) nocturnal 10% hypoxia (N10%; *n* = 7), and (c) continuous 10% hypoxia (C10%; *n* = 8) and (d) corresponding mean ± s.e.m. area under the glucose curve.

Lean control mice on room air exhibited reproducible GTT and ITT curves at the beginning and end of the two-week exposure period (Figures 1(a) and 2(a)). Overnight fasting blood glucose was slightly lower for N10%, but not C10%, exposure on day 14 compared to day 0 (Figures 1(b) and 1(c); time 0), and there was a dose-dependent effect of N10% and C10% hypoxia on improving glucose tolerance in lean mice (Figure 1(d)). In contrast, 2 hr fasting blood glucose was reduced by more than 20 mg/dl for both hypoxic exposures in lean mice immediately prior to the ITT (Figures 2(b) and 2(c); time 0; *p* < 0.025), but the area above the curve was unaffected by both N10% and C10% exposures (Figure 2(d)). Thus, N10% and C10% exposures improved glucose tolerance without evidence of improved insulin tolerance, and in N10%-exposed mice, the metabolic changes occurred independent of weight loss (Table 1).

**Figure 3:**
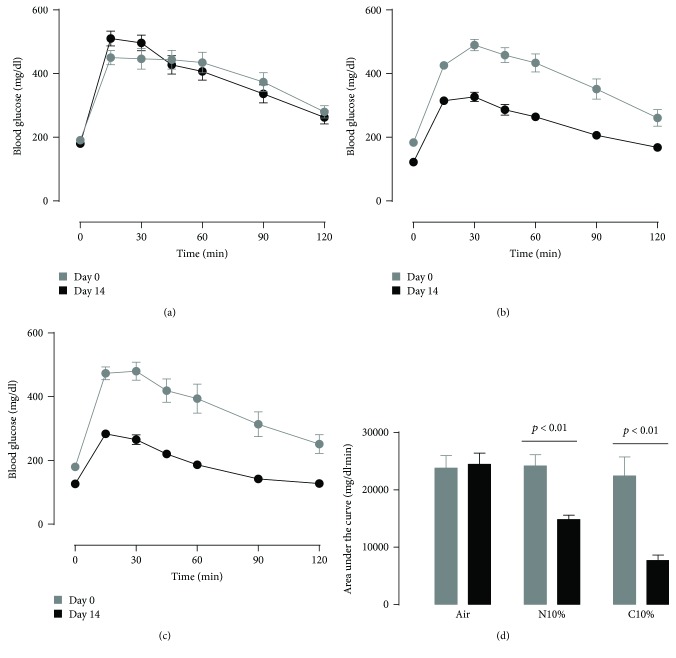
Obese mice: intraperitoneal glucose tolerance test on day 0 (before) and day 14 (after) exposure to two weeks of (a) room air (Air; *n* = 7), (b) nocturnal 10% hypoxia (N10%; *n* = 9), and (c) continuous 10% hypoxia (C10%; *n* = 11), and (d) corresponding mean ± s.e.m. area above the glucose curve. Statistical differences marked by horizontal lines determined by two-tailed paired *t*-test.

**Figure 4:**
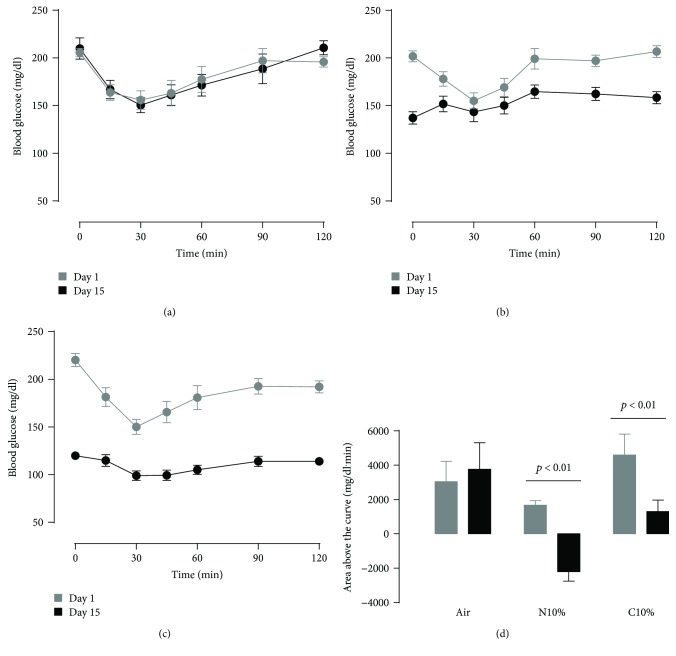
Obese mice: intraperitoneal insulin tolerance test on day 0 (before) and day 14 (after) exposure to two weeks of (a) room air (Air; *n* = 7), (b) nocturnal 10% hypoxia (N10%; *n* = 9), and (c) continuous 10% hypoxia (C10%; *n* = 11) and (d) corresponding mean ± s.e.m. area under the glucose curve. Statistical differences marked by horizontal lines determined by two-tailed paired *t*-test.

Obese mice, prior to hypoxic exposure, exhibited higher overnight fasting blood glucose (Figures 3(a)–3(c); time 0 at day 0; *p* < 0.001) and worse glucose tolerance (Figure 3(d); two left-hand bars; *p* < 0.001) compared to lean mice (Figures 1(a)–1(d)). Similar to lean mice, obese mice showed a dose-dependent effect of N10% and C10% hypoxia on improving glucose tolerance (Figure 3(d)). After both hypoxic exposures, the overnight fasting blood glucose prior to the GTT and the ITT was markedly reduced; for C10% exposure, there was a 53 ± 7 mg/dl reduction at time 0 of the GTT (Figure 3(c); *p* < 0.001) and 100 ± 7 mg/dl reduction at time 0 of the ITT (Figure 4(c); *p* < 0.001). Control obese mice under room air conditions showed a reproducible ITT response (Figure 4(a)). In obese mice exposed to either N10% or C10% (Figures 4(b) and 4(c)), the area above the curve for the ITT was reduced at day 14 compared to preexposure on day 0 (Figure 4(d)). Therefore, in obese mice, exposure to N10% improved glucose intolerance, in the absence of any discernable improvement in insulin tolerance and under conditions of weight neutrality (Table 1).

### 3.2. Obesity Decreases Mitochondrial Efficiency in the Muscle and Increases ROS Production in the Liver

**Figure 5:**
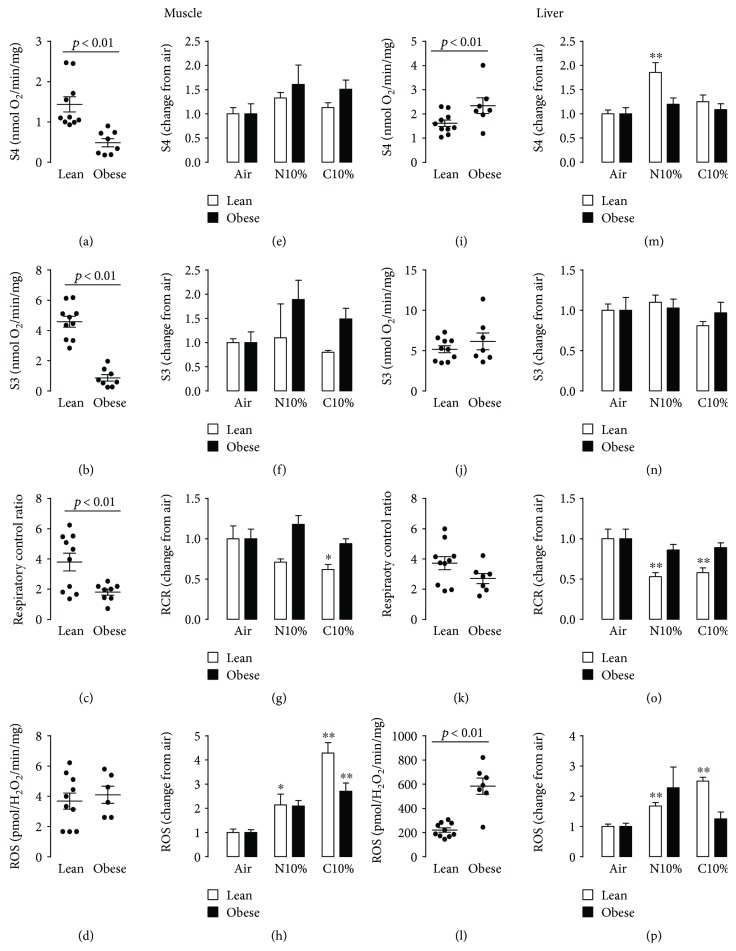
Mitochondrial state 3 (S3), state 4 (S4), and respiratory control ratio (RCR: S3/S4) oxygen consumption and mitochondrial hydrogen peroxide production (reactive oxygen species; ROS) in lean and obese mice. (a–d) Muscle data from individual lean and obese control mice exposed to room air. (e–h) Mean ± s.e.m. change in muscle S3, S4, RCR, and ROS from room air control (Air) in lean and obese mice exposed to nocturnal 10% hypoxia (N10%) and continuous 10% hypoxia (C10%). (i–l) Liver data from individual lean and obese control mice exposed to room air. (m–p) Mean ± s.e.m. change in liver S3, S4, RCR, and ROS from room air control (Air) in lean (*n* = 7–11 per experimental group) and obese mice (*n* = 7–11 per experimental group) exposed to nocturnal 10% hypoxia (N10%) and continuous 10% hypoxia (C10%). Statistical differences marked by horizontal lines determined by two-tailed unpaired *t*-test. Tukey's post hoc analysis of one-way ANOVA used to determine differences relative between Air and either N10% or C10% exposure separately in lean and obese mice. ^∗^*p* < 0.05 compared to Air, ^∗∗^*p* < 0.01 compared to Air.

The obese control room air mice exhibited lower S4, S3, and RCR in the muscle compared to lean mice (Figures 5(a)–5(c)). The ROS levels in the muscle were low and did not differ between lean and obese mice (Figure 5(d)). In the liver under room air conditions, a different effect of obesity was observed (Figures 5(i)–5)(l): S4 was higher in obese mice and S3 and RCR were not different, but liver ROS levels were more than doubled in obese mice compared to lean mice and overall at least 50-fold higher than comparable ROS levels in the muscle.

### 3.3. Hypoxia Decreases Mitochondrial Efficiency and Increases ROS Production in the Muscle and Liver in Lean Mice

In the muscle, exposure to C10% (Figures 5(e)–5(h)) caused a significant reduction in the RCR in lean mice (Figure 5(g); white bars), accompanied by significant increases in ROS production (Figure 5(h); white bars). In contrast, in obese, the RCR was not reduced in response to hypoxia (Figure 5(g); black bars), but did show an increase in ROS production (Figure 5(h); black bars). A similar pattern of response as seen in the muscle from lean mice was evident in the liver (Figures 5(m)–5(p)), with hypoxia causing a decrease in RCR (Figure 5(o); white bars) and an increase in ROS production (Figure 5(p); white bars). In obese mice, there were no significant changes in either RCR (Figure 5(o); black bars) or ROS production (Figure 5(p); black bars) with hypoxic exposure.

### 3.4. Obesity, but Not Hypoxia, Increases GLUT1 Protein Expression

**Figure 6:**
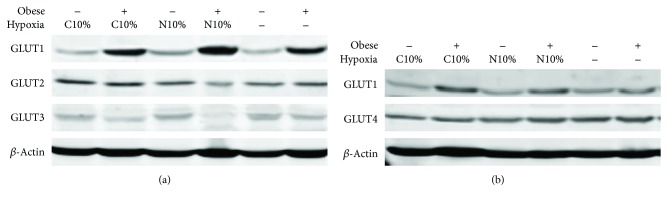
Glucose transporter (GLUT1–4) expression by Western blot after two-week exposure to room air (Hypoxia), nocturnal 10% hypoxia (N10%), and continuous 10% hypoxia (C10%) in lean and obese mice.

GLUT1 expression was increased in obese mice in both muscle and liver, whereas GLUT4 expression in the muscle and GLUT2 and GLUT 3 expression in the liver were not different between lean and obese mice (Figures 6(a) and 6(b) and Table 2). There was no independent effect of hypoxic exposure on any of the glucose transporters in either lean or obese mice (Figures 6(a) and 6(b), Table 2).

### 3.5. N10%, but Not C10%, Increases Glycolytic Activity in the Muscle and Liver in Lean Mice

**Figure 7:**
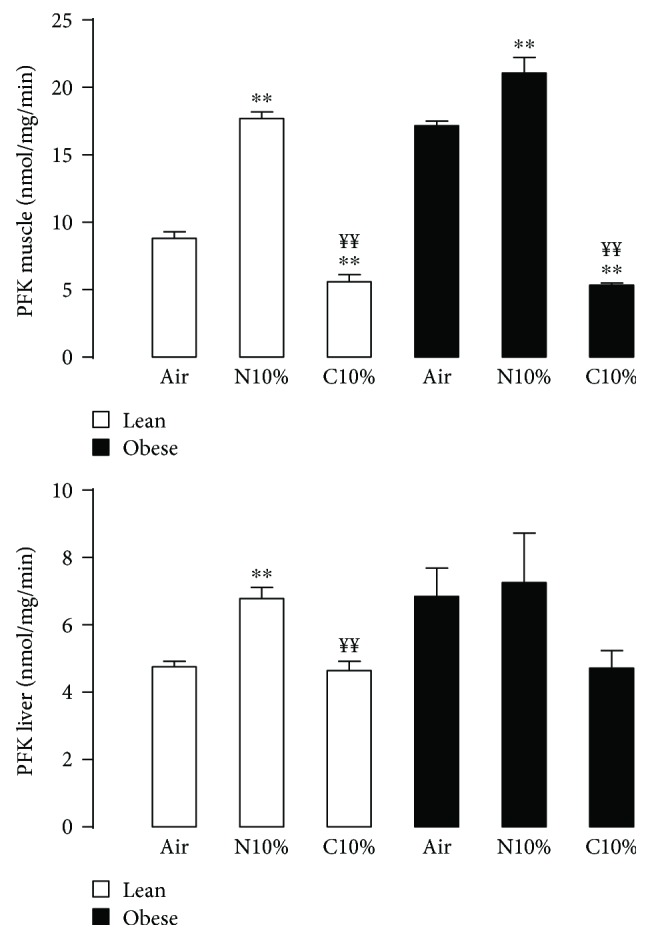
Phosphofructokinase (PFK) activity from the muscle (a) and liver (b) in lean (white; *n* = 4 per experimental group) and obese (black; (*n* = 4 per experimental group) mice after two-week exposure to room air (Air), nocturnal 10% hypoxia (N10%), and continuous 10% hypoxia (C10%). Data shown as mean ± s.e.m. and Tukey's post hoc analysis of one-way ANOVA used to determine differences relative to Air (^∗∗^*p* < 0.01) or N10% (^¥¥^*p* < 0.01) exposure separately in lean and obese mice.

In lean mice, PFK activity in the muscle and liver was increased in mice exposed to N10% compared to room air control mice (Figures 7(a) and 7(b); two left-hand white bars). Interestingly, PFK activity was decreased in lean mice exposed to C10% compared to lean mice exposed to N10%, such that activity was either at or below that seen control mice (Figures 7(a) and 7(b); white bars).

Obese room air control mice had higher PFK activity than lean mice in both muscle (Figure 7(a); *p* < 0.001) and liver (Figure 7(b); *p* < 0.05). Although qualitatively obese mice displayed a similar pattern of response to N10% and C10% exposure as seen in lean mice, only the PFK activity in the muscle was increased with N10% compared to room air control and decreased with C10% compared to N10%.

### 3.6. Hypoxia Increases Lactate in Lean Mice whereas FFA Were Unchanged

For plasma lactate, there was a significant interaction between hypoxia and obesity, with hypoxia increasing plasma lactate in lean mice relative to obese mice (Table 1; *p* < 0.05). In contrast, plasma FFA levels were comparable between the lean and obese mice and did not change with hypoxia.

## 4. Discussion

Hypoxia is a source of systemic, tissue, and cellular stress and when experienced chronically can induce adaptive responses. Here, we show that two weeks of either nocturnal or sustained hypoxia significantly improves glucose disposal in both lean and obese mice without evidence of increased insulin responsiveness. Importantly, the improved glucose disposal with nocturnal hypoxic exposure occurred independent of weight loss. A major goal of our study was to assess the impact of a two-week period of hypoxic exposure on mitochondrial function and ROS production. We found that lean mice exhibited a decrease in RCR, or mitochondrial efficiency, and an increase in ROS production in both muscle and liver and that in combination with increased PFK activity could contribute to improving glucose disposal. In contrast, in obese mice, adaptive decreases in RCR were evident prior to hypoxic exposure, suggesting that reduced mitochondrial efficiency is unlikely to act as a metabolic adaptation contributing to hypoxic-induced improvements in glucose disposal in the presence of obesity. In the discussion that follows, we examine the impact of hypoxia and obesity on adaptations in mitochondrial function and ROS production, upregulation of glucose transporters, and activity of the key glycolytic enzyme PFK in the muscle and liver tissue.

Nocturnal hypoxic exposure in lean mice improved glucose tolerance despite a small but significant increase in body weight. In both liver and muscle of lean mice, nocturnal hypoxia induced a decrease in mitochondrial RCR. Assuming a required amount of ATP production for basal metabolism, any decrease in mitochondrial efficiency, as detailed by a reduced RCR, would necessitate an increase in oxidative metabolism to maintain homeostatic ATP generation. Thus, a decrease in RCR in lean mice exposed to nocturnal hypoxia is consistent with our observed increase in glucose disposal. It appears unlikely that the concomitant increase in ROS production in the muscle and liver resulting from mitochondrial inefficiency negatively impacted glucose disposal. Our data do not support nocturnal hypoxia increasing insulin responsiveness based on the ITT curves, and GLUT4 was not increased in the muscle suggesting the improved glucose disposal was likely more dependent on the established insulin-independent hypoxic-AMPK activation [30–32]; however, it should be noted that GLUT4 was measured under basal, and not insulin-stimulated, conditions. Lastly, increased glucose uptake from nocturnal hypoxia may in part be dependent on our observed augmentation of PFK activity in both muscle and liver. PFK is the rate-limiting step in glycolysis and similar to AMPK is activated by an increased AMP/ATP ratio, which as noted above can result from hypoxia. An increase in glycolysis in lean mice is supported by the elevated lactate levels observed in response to hypoxia. Thus, nocturnal 10% hypoxia in lean mice induces mitochondrial inefficiency, activation of glycolytic enzymes, and improves glucose disposal under conditions of slight weight gain.

With exposure to continuous 10% hypoxia in lean mice, the effects of weight loss on glucose disposal appear to dominate over the effects of hypoxia. Blood glucose prior to the GTT (overnight fast) and ITT (2 hr fast) was significantly lower in C10%-exposed mice suggesting reduced hepatic glucose production and increased muscle glucose disposal, respectively. Mitochondrial efficiency with C10% exposure was reduced comparably to N10% exposure, but associated with higher ROS production. Interestingly, the muscle and liver PFK activity after C10% were reduced compared to N10%, with absolute activity levels at or below that seen in lean room air control mice. It is possible that the high lactate levels after C10% could have contributed to an inhibition of PFK activity [33]. Thus, any potential negative impact of ROS production and attenuated PFK activity on glucose disposal in response to C10% exposure is likely overwhelmed by the impact of weight loss.

In the absence of hypoxia, obese mice exhibited a significant decrease in mitochondrial efficiency in the muscle and a trend to decrease mitochondrial efficiency in the liver compared to lean mice. Similar to the scenario outlined above for responses to hypoxia in lean mice, the decrease in basal RCR in obese mice may represent an adaptive response to improve glucose disposal by uncoupling oxidative phosphorylation and ATP production [34, 35]. Of note, ROS production in the liver of obese mice was almost three times higher than that in lean mice, despite only a nonsignificant trend for reduction in liver RCR, suggesting that liver ROS production may be more dependent on nonmitochondrial sources in obese mice compared to lean mice. Alternatively, a modest but insignificant reduction in liver RCR in obese mice may translate to increased ROS due to increased absolute rates of mitochondrial respiration in the liver compared with the muscle. We also observed that obese mice exhibited elevated GLUT1 protein expression and higher muscle and liver PFK activity than lean mice. Taken together, the diet-induced obese phenotype is characterized by multiple adaptations favoring glucose disposal. However, the combined influence of these obesity-related adaptations was not sufficient to counteract basal hyperglycemia and impaired glucose disposal in mice fed a high-fat diet over a 24-week period.

Hypoxic exposure in obese mice produced marked improvements in glucose tolerance, and similar to lean mice, the response to N10% occurred in the absence of weight loss. However, unlike lean mice, we did not see a decrease in the RCR in obese mice exposed to hypoxia, suggesting that the decrease in basal RCR, discussed above as a potential adaptive response to obesity, may result in a floor effect with hypoxia unable to further reduce mitochondrial efficiency. The pattern of PFK activity in response to hypoxia in obese mice was qualitatively similar to lean mice, although only changes in the muscle PFK activity reached statistical significance. Thus, hypoxia produced similar improvements in glucose disposal in obese and lean mice, but any contribution of reduced mitochondrial efficiency as an adaptive hypoxic response is likely limited to lean mice because the RCR is already reduced in obese mice.

Several points regarding our experimental approach require consideration. The weight loss associated with two weeks of C10% exposure resulted in the mice on days 14 and 15 receiving less glucose and insulin on a weight-dependent basis for the GTT and ITT, respectively. The reduced dosing could have contributed to the decreased area under the curve for the GTT and a decreased area above the curve for the ITT with C10% exposure. However, the improved glucose disposal for N10% exposure was independent of weight change and, therefore, the bolus of glucose and insulin administered. We did not age-match the lean mice to the obese mice; if the mice on a regular chow diet had been age-matched to the high-fat-fed mice, they would have modeled an “overweight” phenotype, given the obesity propensity of the C57BL/6J strain [36]. For the ITT curves, the hypoxic mice on day 15 had significantly lower time 0 blood glucose, particularly the obese mice. Our interpretation of these time 0 data is that the two-hour fast prior to the ITT represents a partial postprandial state in which the hypoxic-adapted mice continue to more rapidly dispose of glucose in a comparable manner to that reflected in the subsequent GTT curves. Thus, for a given postprandial state, the hypoxic mice exhibit a more rapid trajectory to reach a fasting blood glucose. It is difficult to make definitive conclusions about insulin responsiveness with the large differences in initial 2 hr fasting blood glucose in hypoxic mice, but the high reproducibility of the GTT and ITT curves between days 1-2 and days 14-15 in control mice exposed to room air supports the integrity of the data and enabled within subject statistical comparisons. Our mitochondrial function data are normalized to total tissue protein content. Consequently, any reduction in mitochondrial content in response to hypoxia, which has been previously reported as unchanged [37] or decreased [1, 38], could potentially attenuate the effect of mitochondria to increase glucose demand in the muscle and liver by reducing RCR.

Alternatively, short-term exposure to altitude hypoxia can induce acute mountain sickness [11], whereas adaptation occurs over days to weeks at altitude [12, 39]. Recently, we have shown that four weeks of sustained hypoxia in lean mice, simulating altitude exposure, improves glucose disposal relative to four weeks of intermittent hypoxia, simulating obstructive sleep apnea [14]. Studies of high-altitude natives provide biologic plausibility for chronic altitude exposure to improve glucose metabolism [15, 16], and, experimentally, ten nights of moderate hypoxic exposure in obese humans improved glucose disposal [17]. It is likely that sustained hypoxic exposure can impact several pathways to increase glucose uptake.

Our findings have relevance to high-altitude physiology where human studies have shown that the effects of hypoxic exposure on glucose homeostasis are, in part, dependent on exposure duration. Rapid ascent to altitude can cause acute hyperglycemia within the first 48 hrs, but blood glucose normalizes after seven days of acclimation [13]. Moreover, high-altitude natives and obese humans exposed to ten nights of hypoxia exhibit improved glucose metabolism [15–17]. Metabolic adaptations can also occur in patients experiencing hypoxic-related diseases, such as chronic obstructive pulmonary disease (COPD) [40]. Potentially, hypoxic adaptations from COPD may, in part, account for the inability of long-term oxygen therapy to improve glucose metabolism [41].

In summary, we show that nocturnal exposure to 10% hypoxia improves glucose tolerance, independent of weight loss or any improvement in insulin-mediated glucose responsiveness, in both lean and obese mice. Nocturnal 10% hypoxia also increases the liver and muscle PFK activity in lean and obese mice. Reductions in mitochondrial efficiency may contribute to adaptive responses to hypoxia in lean mice and adaptive responses to adiposity in response to a high-fat diet. One potential implication of our study is that a targeted and appropriately titrated hypoxic therapy could potentially improve glucose homeostasis in obese insulin-resistant patients that are free of comorbid hypoxic disease.

## Figures and Tables

**Table 1:** Weight change, food intake, plasma lactate, and plasma free fatty acids (FFA) over two-week exposure to room air (Air), nocturnal 10% hypoxia (N10%), and continuous 10% hypoxia (C10%) in lean and obese mice. Data shown as mean ± s.e.m. and statistical differences between day 0 and day 14 were determined by two-tailed paired *t*-test. Two-way ANOVA for lactate showed a significant effect of hypoxia (*F*(2,41) = 4.3; *p* < 0.05) and obesity (*F*(1,41) = 34; *p* < 0.001) and a significant interaction between hypoxia and obesity (*F*(2,41) = 3.3; *p* < 0.05).

		Air	N10%	C10%
Weight day 0 (g)	Lean	26.0 ± 0.6	24.7 ± 0.6	24.6 ± 0.5
	Obese	46.7 ± 1.6	46.9 ± 1.2	43.8 ± 1.5
Weight day 14 (g)	Lean	26.2 ± 0.5	25.8 ± 0.6	24.0 ± 0.4
	Obese	48.6 ± 1.7	47.1 ± 1.2	38.5 ± 1.8
Weight change (g)	Lean	0.3 ± 0.3	1.1 ± 0.2^∗^	−0.6 ± 0.2^∗^
	Obese	1.9 ± 0.7^∗^	0.1 ± 0.1	−5.3 ± 0.6^∗^
Weight change (%)	Lean	1.1 ± 1.0	4.3 ± 0.8	−2.4 ± 0.8
	Obese	4.2 ± 1.5	0.3 ± 0.4	−12.4 ± 1.6
Food intake (kcal/day)	Lean	10.5 ± 0.5	9.1 ± 0.3	8.3 ± 0.1
	Obese	13.4 ± 0.5	11.7 ± 0.2	8.2 ± 0.2
Lactate (nM)	Lean	4.1 ± 0.4	5.6 ± 0.6	6.0 ± 0.5
	Obese	3.6 ± 0.4	3.4 ± 0.2	3.8 ± 0.01
FFA (mM)	Lean	0.30 ± 0.05	0.22 ± 0.03	0.23 ± 0.01
	Obese	0.33 ± 0.07	0.24 ± 0.02	0.24 ± 0.02

**Table 2:** Glucose transporter (GLUT1–4) expression relative to *β*-actin in the muscle and liver after two-week exposure to room air (Air), nocturnal 10% hypoxia (N10%), and continuous 10% hypoxia (C10%) in lean and obese mice. Data shown as mean ± s.e.m. ^∗^Two-way ANOVA showed increased GLUT1 expression for obese mice relative to lean mice in both muscle (*F*(1,12) = 13.4; *p* < 0.01) and liver (*F*(1,12) = 10.2; *p* < 0.01).

			Air	N10%	C10%
Muscle	GLUT1	Lean	0.32 ± 0.04	0.27 ± 0.03	0.28 ± 0.02
		Obese^∗^	0.33 ± 0.04	0.48 ± 0.04	0.45 ± 0.03
	GLUT4	Lean	0.42 ± 0.02	0.39 ± 0.01	0.43 ± 0.04
		Obese	0.34 ± 0.03	0.38 ± 0.03	0.39 ± 0.04

Liver	GLUT1	Lean	0.31 ± 0.02	0.32 ± 0.05	0.29 ± 0.02
		Obese^∗^	0.51 ± 0.11	0.60 ± 0.11	0.54 ± 0.09
	GLUT2	Lean	0.50 ± 0.04	0.46 ± 0.06	0.51 ± 0.07
		Obese	0.47 ± 0.01	0.37 ± 0.01	0.44 ± 0.07
	GLUT3	Lean	0.71 ± 0.14	0.70 ± 0.13	0.70 ± 0.16
		Obese	0.64 ± 0.15	0.57 ± 0.15	0.52 ± 0.08
